# Wallerian degeneration of bilateral cerebral peduncles after acute carbon monoxide poisoning

**DOI:** 10.1186/s12883-020-01677-5

**Published:** 2020-03-17

**Authors:** Sui-yi Xu, Chang-xin Li, Le-yi Li, Yu Song, Yi Sui

**Affiliations:** 1 Department of Neurology, the First Hospital of Shanxi Medical University, Taiyuan, China; 2 Department of Neurology, the First Affiliated Hospital of Shenzhen University, Health Science Center, Shenzhen Second People’s Hospital, Shenzhen, China; 3 Department of Radiology, Liaoning Jinqiu Hospital, Liaoning Provincial Geriatric Hospital, Shenyang, China; 4 Department of Neurology and Neuroscience, Shenyang First People’s Hospital, Shenyang Brain Hospital, Shenyang Brain Institute, Shenyang Medical College, Shenyang, China

**Keywords:** Wallerian degeneration, Cerebral peduncles, Carbon monoxide, Delayed encephalopathy

## Abstract

**Background:**

Cases of Wallerian degeneration of bilateral cerebral peduncles after acute carbon monoxide poisoning have not yet been reported. To date, most of the delayed encephalopathy after acute carbon monoxide poisoning (DEACMP) lesions captured in magnetic resonance imaging (MRI) has been located in the subcortical white matter and basal ganglia. Here we report two cases of DEACMP with abnormalities in the bilateral cerebral peduncles. The etiology of abnormalities, which were strictly confined to the bilateral cerebral peduncles, was Wallerian degeneration secondary to upstream nerve axonal damage, making this the first report on such bilateral cerebral peduncle abnormalities after DEACMP.

**Case presentation:**

In this report, we present two cases of DEACMP with abnormal signals in the bilateral cerebral peduncles captured during brain MRIs. Case 1 was of a 68-year-old man who presented with paroxysmal disturbance of the consciousness, left limb weakness for 16 days, and lagging responses for 2 days. Case 2 was of a 55-year-old man who was unconscious for 6 h. In addition to the above mentioned characteristics on the brain MRIs, the electroencephalography of case 1 indicated that his forehead scans had a mixture of wide sharp, sharp, and three-phase waves. Brain diffusion tensor imaging of case 2 further proved that the bilateral cerebral anomalies represented Wallerian degeneration secondary to upstream axonal damage. After the definitive diagnosis, the patients returned to the local hospital for hyperbaric oxygen therapy.

**Conclusions:**

Wallerian degeneration of the bilateral cerebral peduncles after acute carbon monoxide poisoning has never been reported before. The abnormal signals in the bilateral cerebral peduncles captured during brain MRIs indicated Wallerian degeneration secondary to upstream axonal damage; thus, these two cases may further our understanding of DEACMP imaging.

## Background

Carbon monoxide (CO) is a colorless and odorless gas. CO poisoning is often caused by improper use of coal stoves for heating and is the cause of the suicide epidemic by charcoal burning in Southeast Asia in recent years [1]. CO poisoning has the dual effect of hypoxia and CO poisoning. Hypoxia itself can cause encephalopathy [2], and neurological damage caused by CO can lead to delayed encephalopathy after acute carbon monoxide poisoning (DEACMP). The diagnostic criterion for DEACMP [3] includes any of the following clinical abnormalities observed 2–60 days after the recovery of the consciousness disorder caused by acute CO poisoning: 1) disturbances of mental state and/or consciousness (such as dementia or delirium), 2) extrapyramidal lesions (such as Parkinson’s syndrome), and 3) pyramidal damage and focal cortical dysfunctions.

Magnetic resonance imaging (MRI) suggests that most DEACMP lesions are located in the subcortical white matter and basal ganglia. Clinical manifestations include cognitive impairment, dyskinesia, forced crying, forced laughter, chorea, and Parkinson’s syndrome [4, 5]. However, bilateral cerebral peduncle anomalies captured on MRIs have not been reported. Herein we report two cases of DEACMP with Wallerian degeneration of the bilateral cerebral peduncles from different medical centers in China.

## Case presentation

### Case 1

The patient was a 68-year-old man who heated his home by burning wood or coal. Sixteen days prior, relatives found the patient lying on the ground in his room, unconscious, accompanied by vomit and incontinence. At the time of discovery, his relatives found that the coal stove was extinguished. He was alert during the brain computed tomography (CT) scan and complained of left limb weakness. The patient was diagnosed with a cerebral infarction and treated with antiplatelets and statin. Two days prior, the patient lagged in responses and would not eat on his own, and was transferred to our hospital. The patient had a prior medical history of prostatic hyperplasia (2 years ago), which had not been treated. He was a drinker and had been consuming alcohol (50 ml, twice a day) for more than 40 years.

On admission, his blood pressure was 140/90 mmHg. He was found to be lagging in responses and had memory deterioration. His left nasolabial groove was shallow. There were no other positive signs of nervous system impairment. On the third day after hospitalization, the patient’s nervous system symptoms worsened. Physical examination revealed that he had difficulty in understanding, expression, memory, character, and spatial orientation. His tongue was slightly to the right. His right proximal lower extremity muscle strength was grade 4+, and his distal muscle strength was grade 3.

No obvious abnormalities were revealed during his emergency brain CT and electrocardiography. His serological analysis data, such as arterial blood carboxyhemoglobin, blood sugar, glycosylated hemoglobin, electrolytes, blood cholesterol, coagulation function, myocardial enzymes, myohemoglobin, troponin, hepatic function, renal function, rheumatism series findings, thyroid function, folacin, vitamin B12, treponema pallidum antibody, hepatitis antigens and antibodies, and human immunodeficiency virus antibody, were found to be within normal limits. Certain laboratory indicators including thyroid stimulating hormone, blood sedimentation, and prostate specific antigens, were slightly elevated, but not specific. Cerebrospinal fluid findings which included sugar, chloride, protein, cell count, acid-fast staining results, ink staining results, autoimmune encephalitis, and paraneoplastic series findings, were normal.

**Fig. 1:**
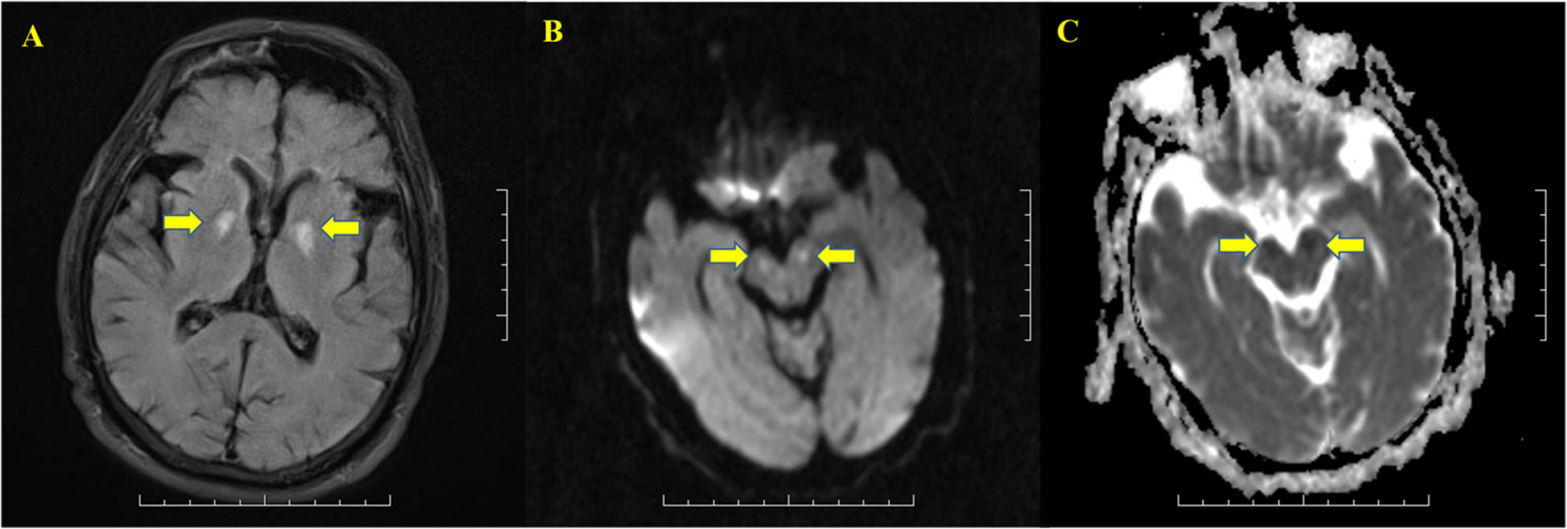
A bilateral symmetrical anomaly signal of the basal ganglia was captured in fluid attenuated inversion recovery images (**a**). Bilateral cerebral peduncles showed symmetrical high signals in DWI (**b**), but low signals in ADC

**Fig. 2:**
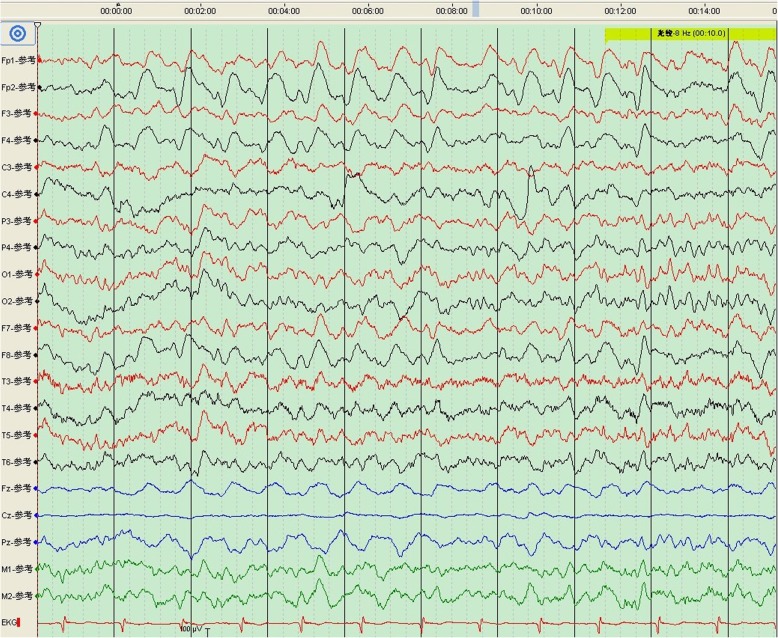
The EEG indicated a diffuse distribution of Delta waves. The Fp1, Fp2, F4 in the frontal lobe area had a mixture of wide sharp, sharp, and three-phase waves. No clinical events occurred during the monitoring process

His MRI showed an abnormal symmetrical signal in the bilateral basal ganglia, which was considered to be indicative of DEACMP (Fig. 1a). Of note, the bilateral cerebral peduncles also showed abnormal symmetrical signals (Fig. 1b-c). The patient’s EEG indicated that Fp1, Fp2, F4, F8 leads in the frontal lobe area had a mixture of wide sharp, sharp, and three-phase waves (Fig. 2). Based on the above clinical manifestations and examination results, the patient was diagnosed with DEACMP. We recommend that the patient be treated with hyperbaric oxygen therapy (HBO). The relatives decided to return the patient to the local hospital to continue rehabilitation. At hospital discharge, the patient had blurred consciousness, indifferent expression, poor understanding, poor memory, poor orientation of characters and space, slow response, and low speech. His tongue was slightly to the right. Proximity muscle strength of right lower limb was grade 4+, and distal muscle strength was grade 3-.

### Case 2

The patient was a 55-year-old man who was unconsciousness for 6 h. The patient lived in the countryside. He heated his home by burning coal. He was found unconscious in a room with the smell of soot. The patient could only answer to simple questions and was brought to our hospital by an ambulance. The patient had a prior medical history of hypertension, gastric ulcers, and diabetes for more than 10 years. He had a suspected history of cerebral infarction for half a year, and his left side limbs were less flexible.

On admission, his blood glucose was 11 mmol/L, and his blood pressure was 129/84 mmHg. He remained unconscious during the physical examination. His limbs moved voluntarily. His muscle tone was normal, and he had no muscle atrophy. Bilateral tendon reflexes could be symmetrically elicited. The Babinski sign and meningeal irritation were negative. His serological parameters which included blood cell findings, electrolytes, B type natriuretic peptide precursors, procalcitonin, liver enzymes, renal function, coagulation function, and D-dimer, were found to be within normal limits. The patient’s glycated hemoglobin and homocysteine levels were above normal.

**Fig. 3:**
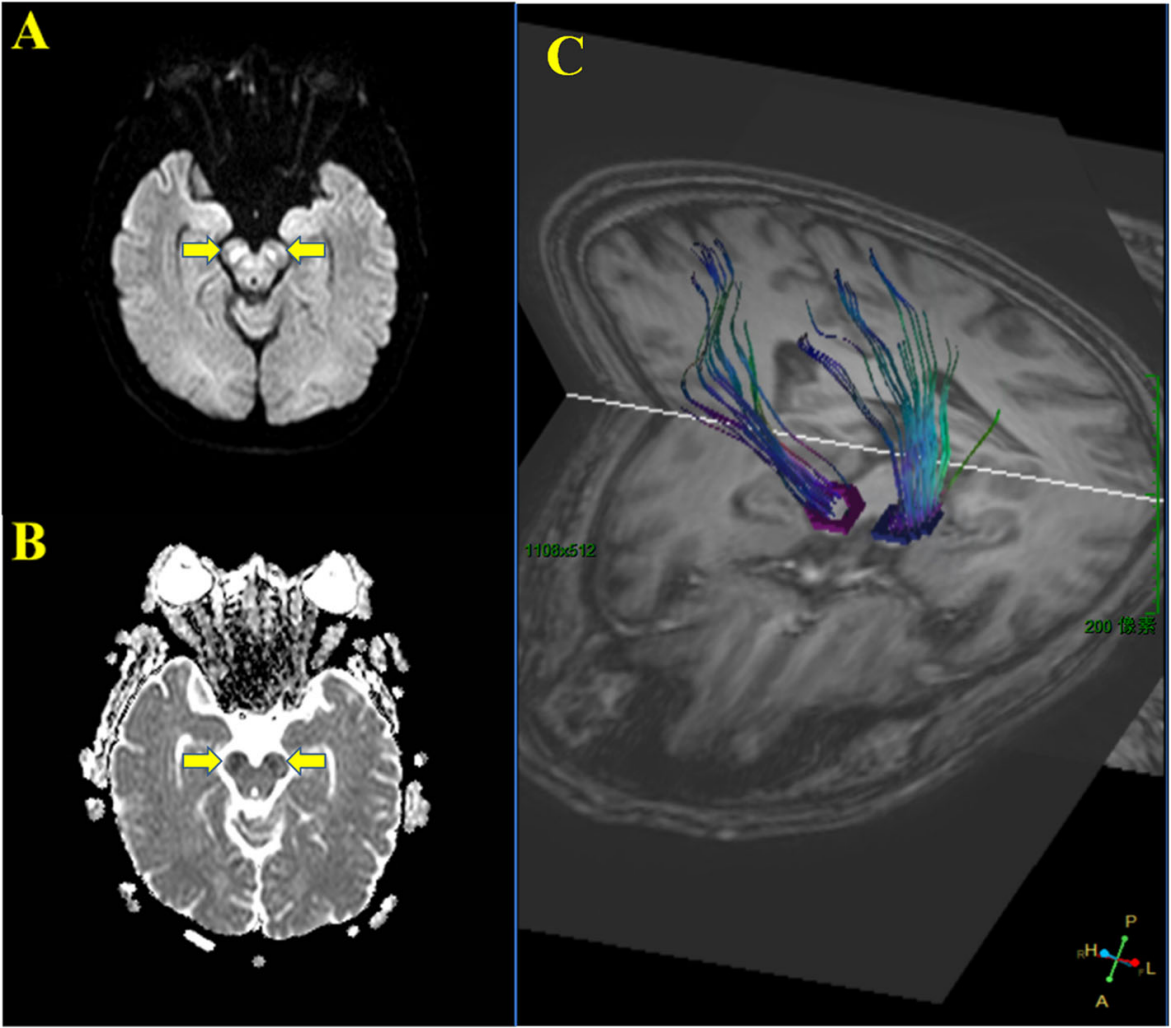
Similar to case 1, bilateral cerebral peduncles showed symmetrical high signals in DWI (**a**), but low signals in ADC (**b**). Wallerian degeneration of bilateral cerebral peduncles secondary to corticospinal tract injury was confirmed by DTI.

**Fig. 4:**
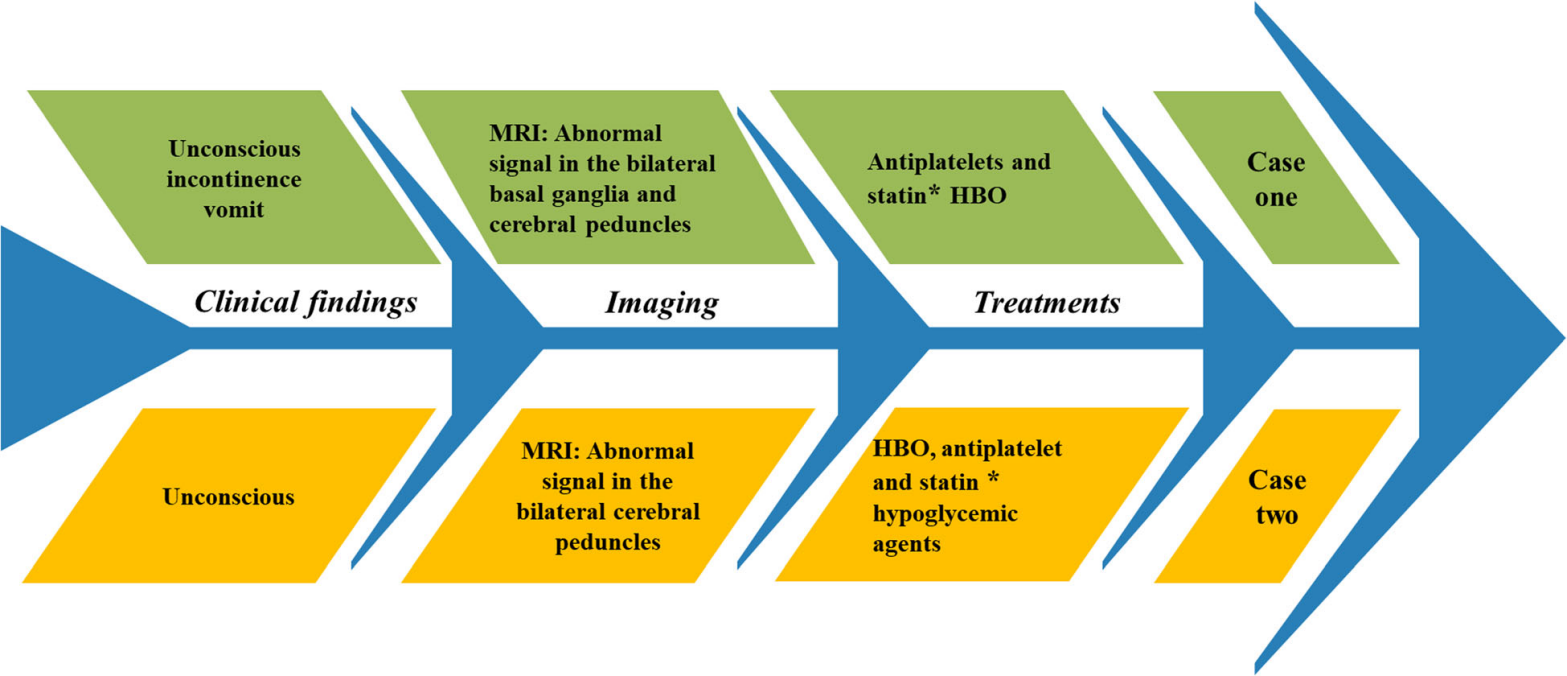
Timeline of the cases. * Case 1 was misdiagnosed as cerebral infarction by the local hospital, and was given antiplatelet and statin treatment. * Case 2 was treated with antiplatelet and statin because of previous stroke history

His brain CT scan showed no obvious abnormalities. The patient was treated with HBO, antiplatelet, statin, and hypoglycemic agents. On the second day after admission, the patient was conscious. Interestingly, similar to case 1, the bilateral cerebral peduncles showed high symmetrical signals on brain diffusion-weighed imaging (DWI) (Fig. 3a), and low signals at corresponding areas in apparent dispersion coefficient (ADC) sequences (Fig. 3b). Diffusion tensor imaging (DTI) confirmed Wallerian degeneration secondary to nerve fiber bundle injury (Fig. 3c). The patient was diagnosed with DEACMP and treated with HBO until discharge from the hospital after 4 weeks. Moreover, the patient was treated with antiplatelet, statin and hypoglycemic agents because of previous stroke and diabetes history (Fig. 4). At hospital discharge, the patient was conscious. His speech was slightly slurred. The muscle strength was grade 4 with increased muscle tone.

## Discussion and conclusions

In this report, we present two cases of abnormal signals in bilateral cerebral peduncles after carbon monoxide poisoning (Fig. 4). Both cases manifested as a disturbance of consciousness, which meet the diagnostic criteria of DEACMP as previously described [3]. However, the differential diagnosis of abnormal signals in bilateral cerebral peduncles in MRI should be identified (Table 1) [6–8]. Especially it needs to be distinguished from ischemic stroke when high signals are present on DWI while low signals occur in ADC. Of note, DWI/ADC lesions do not necessarily mean the occurrence of Wallerian degeneration. The restricted diffusion of white matter lesion might indicate slow and progressive development of cellular edema as a result of neuronal death and delayed demyelination in DEACMP [9]. In the case two patient, DTI was used to confirm that the abnormal signals of bilateral cerebral peduncles were Wallerian degeneration or just the sign of DEACMP.

**Table 1:** The differential diagnosis of bilateral cerebral peduncles abnormity

Author	Date	Subject	Lesion	Etiology	Imaging
Waragai [6]	1994	Human	Cerebral hemisphere	Stroke	MRI, T_2_ image
Zakaria [7]	2006	Human	Basilar artery posterior cerebral arteries	Stroke	DWI, MRA
Tuor [8]	2013	Neonatal rat	Cerebral hemisphere	Hypoxia ischemia	MRI, T_2_ image ADC

Although basal ganglion necrosis is often mentioned in the literature for carbon monoxide poisoning, the incidence of globus pallidus lesions is significantly lower than that of white matter lesions [1]. As early as 1982 [10], some scholars found that early white matter lesions in carbon monoxide poisoning were neither true demyelination nor selective involvement of oligodendrocytes, but rather axonal damage with glial changes followed by Wallerian degeneration. Glial activation may play an important role in DEACMP [11], with serum S100B or glial fibrillary acidic protein levels being related to the prognosis. A recent study reported [12] that demyelination by CO is more severe than that by simple hypoxia, and the former lasts longer.

At present, there are few EEG reports for DEACMP. In Case 1 reported here, the patient’s EEG indicated that Fp1, Fp2, F4, F8 leads in the frontal lobe area had a mixture of wide sharp, sharp, and three-phase waves which implied forehead cortical dysfunction. In a case report regarding generalized chorea caused by DEACMP [5], the patient’s EEG showed a poorly persistent alpha background and extensive slow waves, mainly in the bilateral frontal and temporal lobes, suggesting cortical dysfunction.

HBO has been a traditional method of treating DEACMP in basic and clinical studies. Dexamethasone combined with HBO therapy has been reported to have better efficacy than HBO alone [13]. Myelin basic protein (MBP) levels in cerebrospinal fluid decreases significantly after treatment. Basic experiments have also confirmed that [14] dexamethasone can inhibit the immune response, preventing MBP degradation and DEACMP in the rat model. Furthermore, methylene blue was shown to be effective in a rat model of DEACMP [15]. Cognitive impairment often occurs in DEACMP [16, 17]. Our case one showed difficulty in understanding, expression, memory, character, and spatial orientation. The mechanism may be related to the affection of nicotinic cholinergic system after DEACMP [18]. Recent studies have confirmed that acetylcholinesterase inhibitors such as donepezil or galanthamine could alleviate cognitive impairment associated with DEACMP. Interestingly, the latter seems to be better than the former [19, 20]. Moreover, compared with HBO treatment alone, the combination of N-butylphthalide and HBO for disease management may significantly improve the mini-mental state examination scores [21, 22].

To our knowledge, this is the first DEACMP report concerning bilateral cerebral peduncle anomalies captured on MRI. We further demonstrated with DTI that this symmetrical anomaly was due to the Wallerian degeneration of bilateral cerebral peduncles secondary to upstream axonal damage. This report may help further our understanding of DEACMP imaging.

## Data Availability

Not applicable.

## Abbreviations

ADC: Apparent dispersion coefficient
CO: Carbon monoxide
CT: Computed tomography
DTI: Diffusion tensor imaging
DEACMP: Delayed encephalopathy after acute carbon monoxide poisoning
EEG: Electroencephalography
GFAP: Glial fibrillary acidic protein
HBO: Hyperbaric oxygen
MBP: Myelin basic protein
MRI: Magnetic resonance imaging
MMSE: Mini-mental state examination
